# Rapid identification of microbial pathogens in intra-abdominal infections using multiplex PCR

**DOI:** 10.3389/fcimb.2026.1879793

**Published:** 2026-07-22

**Authors:** Johanna Bückner, Uwe Groß, Nils Kunze-Szikszay, Michael Ghadimi, Konrad Meissner, Onnen Moerer, Thorsten Perl

**Affiliations:** 1 Department of General, Visceral, and Pediatric Surgery, University Medical Center Göttingen, Göttingen, Germany; 2 Institute for Medical Microbiology and Virology, University Medical Center Göttingen, Göttingen, Germany; 3 Department of Anesthesiology, University Medical Center Göttingen, Göttingen, Germany

**Keywords:** intra-abdominal infection, microbiological diagnostic techniques, multiplex PCR, peritonitis, point-of-care-diagnostic, polymicrobial infection, rapid pathogen detection

## Abstract

Intra-abdominal infections (IAI) resulting from the perforation of hollow viscera are associated with high morbidity and mortality. In addition to timely surgical source control, rapid and accurate pathogen identification is crucial to guide targeted antimicrobial therapy, particularly in polymicrobial infections involving potentially virulent pathogens. In this prospective study (ClinicalTrials.gov NCT07562204), we compared real-time multiplex PCR Unyvero A50 (Curetis^®^) as a point-of-care (POC) tool with conventional microbiological culture (cMB). 50 patients with suspected hollow organ perforation or peritonitis were included. 2 intraoperative samples were collected per patient: one for immediate PCR testing and one for standard culture-based diagnostics. Turnaround times, pathogen detection rates, resistance marker detections and microbial distribution according to anatomical sampling site were assessed descriptively. PCR results were available on the same day or the next day in all cases (n=41), whereas cMB results required *≥*2 days in all cases (n=50). 9 samples (18%) yielded invalid PCR results according to the manufacturer’s system output and were excluded from concordance analyses. Among the 41 evaluable sample pairs, complete concordance between PCR and cMB was observed in 29% (12/41), while non-concordant results occurred in 71% (29/41). PCR detected at least one additional pathogen not identified by cMB in 49% (20/41) of cases. Pathogen detection rates and pathogen group distributions were analyzed per protocol in samples with valid PCR results (n=41) and compared with cMB results from the total cohort (n=50). Gram-positive bacteria were detected in 58% (29/50) of cMB and 56% (23/41) of PCR samples. Aerobic/facultative Gram-negative bacteria were detected in 46% (23/50) of cMB samples and in 59% (24/41) of PCR samples. *Enterobacteriaceae* represented the predominant subgroup and were detected in 34% (17/50) of cMB and 37% (15/41) of PCR samples. Anaerobic bacteria were detected more frequently by PCR than by cMB (cMB 24%, 12/50 vs. PCR 44%, 18/41). In conclusion, multiplex PCR cannot replace conventional culture, but may serve as a complementary tool, providing rapid detection of selected pathogens, including anaerobes. Its implementation may support earlier antimicrobial decision-making in IAIs. Further multicenter studies are needed to evaluate its clinical utility in routine diagnostic workflows.

## 1 Introduction

Sepsis secondary to hollow viscus perforation is associated with high morbidity and mortality rates (Arvaniti et al., 2022; Prescott et al., 2026). Such perforations may arise from infections, malignancies, peptic ulcers, or iatrogenic injuries and frequently result in intra-abdominal infections (IAIs) or peritonitis – conditions that can rapidly progress and become life-threatening if not diagnosed and treated promptly (Doklestic et al., 2014; De Pascale et al., 2022). This rapid clinical deterioration is partly driven by disruption of the mucosal barrier and subsequent translocation of pathogens into the peritoneal cavity. Effective management of IAIs requires prompt surgical source control combined with early initiation of appropriate antimicrobial therapy (Eckmann et al., 2020; Sartelli et al., 2024).

The interval between clinical deterioration and the initiation of effective antimicrobial therapy is a key determinant of patient outcome. Kumar et al. (Kumar et al., 2006) and Li et al. (Li et al., 2026) demonstrated strong associations between delays in antimicrobial therapy initiation and increased mortality in critically ill patients with septic shock. In the absence of rapid diagnostic results, empiric broad-spectrum antimicrobial therapy remains standard practice, which may lead to inappropriate treatment, increased toxicity, and the emergence of antimicrobial resistance (Labricciosa et al., 2018; Sartelli et al., 2024). These challenges underscore the urgent need for fast and reliable diagnostic tools in the management of IAIs.

Furthermore, faster diagnostic turnaround times may facilitate earlier reassessment of antimicrobial therapy and may contribute to reduced antimicrobial exposure or resource utilization when integrated into appropriate clinical workflows (Edelsberg et al., 2008; Chong et al., 2015).

Routine microbiological diagnostic methods rely on culture-based techniques, which typically require 48 hours or longer for pathogen identification and susceptibility testing. In contrast, newer molecular approaches – such as nucleic acid amplification tests, including polymerase chain reaction (PCR) – offer substantially shorter turnaround times and may enable earlier pathogen-directed therapy (Liesenfeld et al., 2014; Kunze et al., 2015; Candel et al., 2024). Real-time multiplex PCR allows simultaneous detection of multiple preselected pathogens and resistance markers directly from clinical samples, although direct assignment of resistance to a defined pathogen may be inconclusive in polymicrobial samples. Compared with conventional culture, it can deliver diagnostic results within a few hours and is feasible as a point-of-care (POC) diagnostic tool. Despite these advantages, such technologies are not yet widely established as standard adjuncts to conventional microbiological culture (cMB) in routine clinical practice (Candel et al., 2024).

Therefore, the present study aims to evaluate the concordance and turnaround time of the multiplex PCR Unyvero A50 system compared with cMB in intra-abdominal samples from patients with perforation peritonitis. In addition, we assess its potential as a rapid POC tool to provide earlier microbiological information that may support antimicrobial reassessment in selected patients. The study was not designed to evaluate PCR-guided treatment changes or clinical outcomes.

## 2 Materials and methods

### 2.1 Study design and patient population

We conducted a prospective observational study including 50 adult patients with suspected hollow organ perforation or peritonitis, admitted to the University Medical Center Göttingen (UMCG) between June 2018 and December 2020. This study was designed as an exploratory prospective observational study to assess the feasibility, turnaround time, and descriptive pathogen detection patterns of multiplex PCR compared with conventional microbiological culture. No formal sample size calculation was performed, as no confirmatory hypothesis testing was planned. The sample size was based on the expected number of eligible patients during the study period, the single-center design, the feasibility of intraoperative paired sampling, and the availability of resources for point-of-care PCR testing. The study protocol was approved by the ethics committee of the Faculty of Medicine, University of Göttingen (approval number 1/7/06, amendment 4) and registered at ClinicalTrials.gov (NCT07562204). Informed consent or deferred consent was obtained from all patients. Patients were included if hollow organ perforation was suspected pre- or intraoperatively and intra-abdominal sampling for microbiological diagnostics was clinically indicated; samples were obtained intraoperatively. Patients in moribund conditions at presentation or already enrolled in an interventional clinical trial were excluded. All included patients underwent surgical treatment and were subsequently admitted to the intensive care unit for postoperative care.

### 2.2 Sample collection and conventional microbiological culture

During surgery, two intra-abdominal peritoneal fluid samples were collected from each patient. One sample was sent immediately to the Institute of Medical Microbiology and Virology, UMCG, for cMB analysis including aerobic and anaerobic culture, pathogen identification using matrix-assisted laser desorption/ionization time-of-flight mass spectrometry (MALDI-TOF MS) (Bruker Daltonics, Bremen, Germany) and antimicrobial susceptibility testing using the VITEK 2 system version 07.01 (bioMérieux, Nürtingen, Germany).

### 2.3 Multiplex PCR testing

The second sample was processed using real-time multiplex PCR postoperatively at the surgical intensive care unit. PCR testing was performed using the Unyvero A50 system (Curetis AG, Holzgerlingen, Germany) with the IAI cartridge, following the manufacturer’s instructions (Curetis, 2017) (cartridge REF 10064). The IAI cartridge detected a predefined panel of bacterial and fungal pathogens, as well as selected resistance and toxin genes commonly associated with intra-abdominal infections. Detection was based on amplification of species- or group-specific genomic targets using real-time PCR, and results were available in four to five hours according to manufacturer’s information.

### 2.4 Turnaround time assessment

Turnaround time was recorded in calendar days rather than hours because time stamps were not consistently available across workflows. Results were categorized as 0 days (same calendar day as sampling), 1 day (next calendar day), or ≥2 days. Tests completed after midnight were assigned to the next-day category (1 day) based on the date of availability of the result.

### 2.5 Interpretation of PCR results and handling of invalid runs

Test results were interpreted according to the manufacturer’s predefined algorithm. A complete list of detectable pathogens, resistance markers, and toxin genes is provided in **Tables 1**–**3**. PCR results were reported qualitatively as positive or negative for each target. Some targets represent genus-level groups (spp.) and may co-occur with species-level targets in the same sample; thus, detection counts may include overlapping targets. In polymicrobial samples, detected resistance genes were not assigned to individual pathogens. Resistance marker detections were analyzed descriptively in samples with valid PCR results and reported together with pathogens detected in the same PCR sample. PCR-based resistance marker detection was interpreted as supplementary information and was not considered equivalent to phenotypic antimicrobial susceptibility testing.

**Table 1:** Pathogens detectable by the Unyvero A50 IAI panel.

Gram-positive bacteria	*Enterobacteriaceae*	Anaerobes/ facultative anaerobes	Non-fermenting bacteria	Universal bacteria	Fungi
*Streptococcus* spp.^1^	*Escherichia coli*	*Aeromonas* spp.^8^	*Acinetobacter baumannii* complex^11^	Detection of prokaryotic genomic sequences^12^	*Candida* spp.^13^
*Staphylococcus aureus*	*Klebsiella aerogenes (E. aerogenes)*	*Bacteroides fragilis* group^9^	*Pseudomonas aeruginosa*		*Candida albicans*
Coagulase-negative *Staphylococcus* spp.^2^	*Enterobacter cloacae* complex^4^	*Bacteroides* spp./*Prevotella* spp.^10^			*Candida glabrata*
*Enterococcus* spp.^3^	*Klebsiella pneumoniae* ^5^	*Clostridium difficile*			*Candida tropicalis*
*Enterococcus faecalis*	*Klebsiella oxytoca*	*Clostridium perfringens*			*Issatchenkia orientalis (Candida krusei)*
	*Klebsiella variicola* ^6^	*Finegoldia magna*			
	*Proteus* spp.^7^	*Propionibacterium acnes*			

^1^S. pneumoniae, S. constellatus, S. anginosus, S. agalactiae, S. pyogenes, S. intermedius.

^2^S. saprophyticus, S. hominis, S. epidermidis, S. warneri, S. haemolyticus, S. capitis, S. lugdunensis.

^3^E. faecalis, E. faecium, E. gallinarium, E. casseliflavus, E. avium, E. hirae, E. durans, E. raffinosus.

^4^E. cloacae, E. asburiae, E. hormaechei.

^5^Klebsiella pneumoniae Cluster I + II (according to Alves, Dias (Alves et al., 2006)), K. pneumoniae subsp. Ozaenae.

^6^Klebsiella variicola (Cluster III).

^7^P. vulgaris, P. mirabilis, P. penneri, P. hauseri.

^8^A. hydrophila subsp. hydrophila, A. sobria, A. media, A. veronii, A. caviae.

^9^B. fragilis, B. ovatus, B. thetaiotaomicron, B. uniformis.

^10^B. fragilis, B. dorei, B. faecis, B. heparinolyticus, B. ovatus, B. pyogenes, B. thetaiotaomicron, B. uniformis, B. vulgatus, Parabacteroides distasonis, P. bivia, P. buccae, P. denticola, P. disiens, P. intermedia, P. jejuni, P. melaninogenica, P. nanceiensis, P. nigrescens, P. oris.

^11^A. baumannii, A. oleivorans, A. pittii.

^12^universal primer for bacterial detection.

^13^C. albicans, C. tropicalis, C. orthopsilosis, C. parapsilosis, C. dubliniensis, C. viswanathii, C. metapsilosis, C. labiduridarum, C. theae as well as Lodderomyces elongisporis, Schwanniomyces etchellsii, Millerozyma farinosa (Pichia farinosa), Schwanniomyces occidentalis, Yamadazyma triangularis (Candida polymorpha), Phaeosphaeria nodorum.

**Table 2:** Toxins detectable by the Unyvero A50 IAI cartridge.

Toxin	Marker	Organism
Toxin B	*tcd*B	*Clostridium difficile*
Shiga toxin	*stx1/stx2*	*Escherichia coli*

**Table 3:** Resistance markers detectable by the Unyvero A50 IAI cartridge.

Antibiotic /class	Resistance gene
Oxacillin	mecA, mecC
Glycopeptides	vanA, vanB
Aminoglycosides	aacA4
Cephalosporin (3rd generation)	ctx-M
Fosfomycin	fosA3
Polypeptides / Polymyxins	mcr-1
Nitroimidazole	nimA, nimB
Fluoroquinolones	qnrA, qnrB, qnrS
Tetracycline	tetA
Carbapenems	imp, kpc, ndm, oxa-23, oxa-24/40, oxa-48, oxa-58, vim

PCR runs yielding invalid results according to the manufacturer’s system output were classified as non-evaluable. These samples were excluded from all PCR-based analyses, including pathogen distribution, number of detected pathogens per sample, and concordance analyses. cMB results from these patients remained included in analyses of the total cMB cohort. Repeat PCR testing was not performed as part of the study protocol. Detailed technical causes of invalid PCR results were not systematically documented, as the system output did not specify the underlying cause of invalidity.

### 2.6 Concordance analysis

Concordance was assessed at two levels. Complete sample-level concordance was defined as identical pathogen detection profiles between cMB and PCR within an evaluable sample pair. In addition, target-level concordance was analyzed descriptively for each pathogen target included in the PCR panel and summarized by pathogen group. Target-level agreement was calculated as the sum of concordant positive and concordant negative target comparisons divided by the total number of target comparisons within the respective pathogen group: (cMB+/PCR+ + cMB−/PCR−)/all target comparisons.

### 2.7 Statistical analysis

Statistical analyses were primarily descriptive, reflecting the exploratory nature of this prospective observational study. Categorical variables were reported as absolute numbers and proportions. Analyses were performed using Microsoft Excel (Microsoft Corporation, Redmond, WA, USA). Summary measures were presented as means with standard deviation where appropriate. No inferential statistical testing was performed.

## 3 Results

### 3.1 Study population and sampling sites

The characteristics of the study population and sampling locations are summarized in **Table 4**. Most intra-abdominal samples originated from upper abdominal sites, which accounted for 72% of the cohort. Within the upper abdominal group, small intestinal and duodenal sources together represented the largest subgroup.

**Table 4:** Clinical characteristics of the study population and anatomical sampling sites.

Variable	Value
Female	18 (36%)
Male	32 (64%)
Age (years)	63.4 ( ± 13.5)
Height (cm)	173.1 ( ± 8.9)
Weight (kg)	76.9 ( ± 16.0)
BMI (kg/m^2^)	25.6 ( ± 4.9)
Length of stay at ICU (days)	9.5 ( ± 16.6)
Length of stay at hospital (days)	33.9 ( ± 24.0)
Upper abdominal localization^1^	36 (72%)
Lower abdominal localization^2^	14 (28%)

^1^Upper abdominal sites include small intestine (18%), stomach (14%), gallbladder/bile ducts (14%), duodenum (12%), pancreas (14%). Percentages refer to the total cohort (n=50).

^2^Lower abdominal/colorectal sites include sigmoid colon (18%), cecum (4%), descending colon (2%), others (4%). Percentages refer to the total cohort (n=50).

Values presented as mean ± standard deviation or number (percentage), as appropriate.

### 3.2 Validity of PCR results and turnaround time

All PCR-based analyses were restricted to samples with valid PCR results (n=41), whereas conventional microbiological culture (cMB) results refer to the total cohort (n=50). Of the 50 PCR runs, 41 yielded valid results and were included in PCR-based analyses, corresponding to a valid PCR rate of 82%. Nine PCR runs (18%) yielded invalid results according to the manufacturer’s system output and were therefore excluded from PCR-based and concordance analyses. cMB results from these patients remained included in the total cMB cohort. The specific technical causes of invalid PCR results were not systematically documented.

Valid multiplex PCR results were available on the same day of surgery in 61% (25/41) of cases and on the following day in 39% (16/41). In contrast, cMB results were reported ≥2 days after sampling in all cases (100%, 50/50).

### 3.3 Pathogen detection and pathogen group distribution

**Figure 1:**
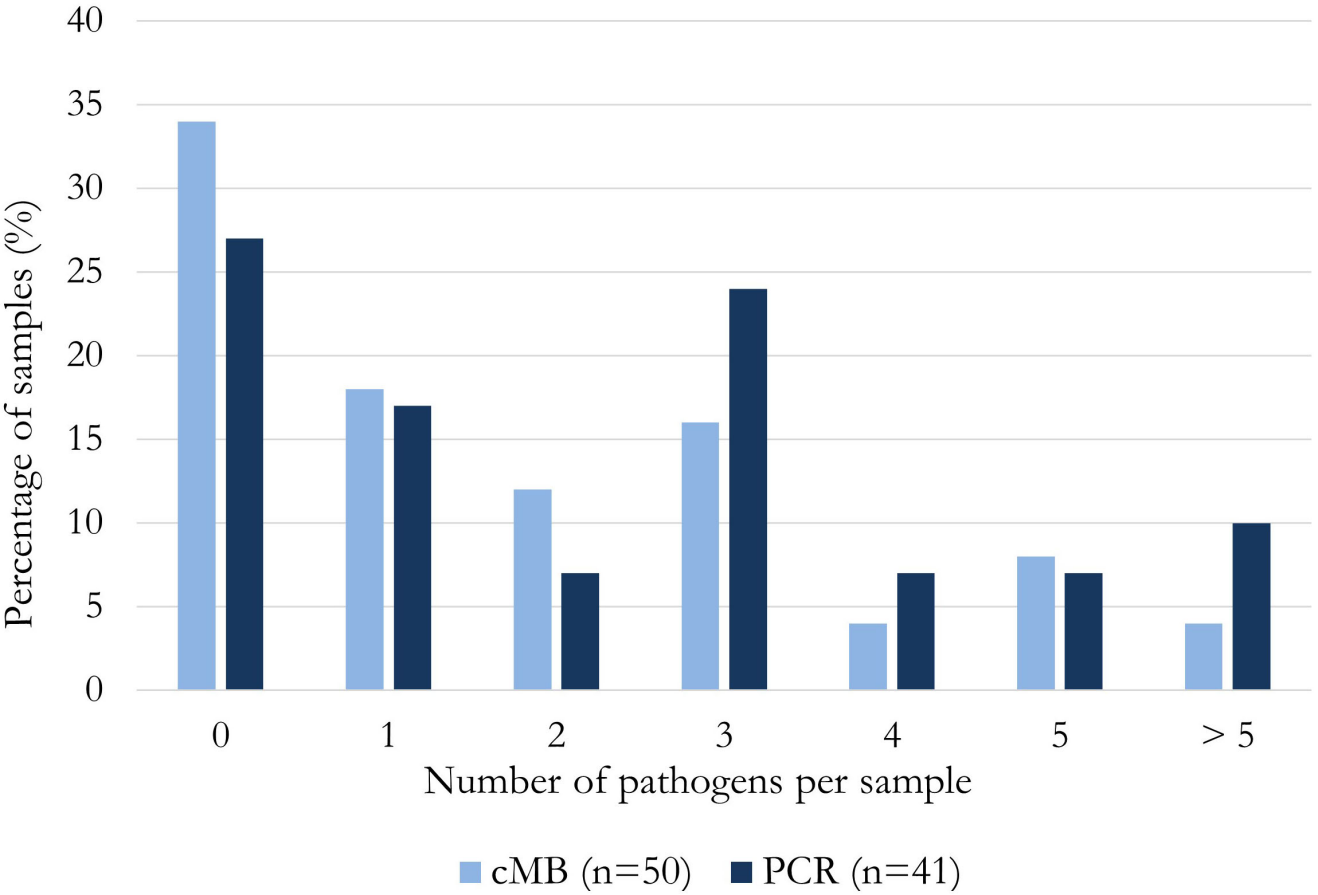
Distribution of detected pathogens per sample by conventional microbiological culture (cMB) and multiplex PCR (PCR), expressed as percentages of samples (cMB n=50, PCR n=41).

**Figure 2:**
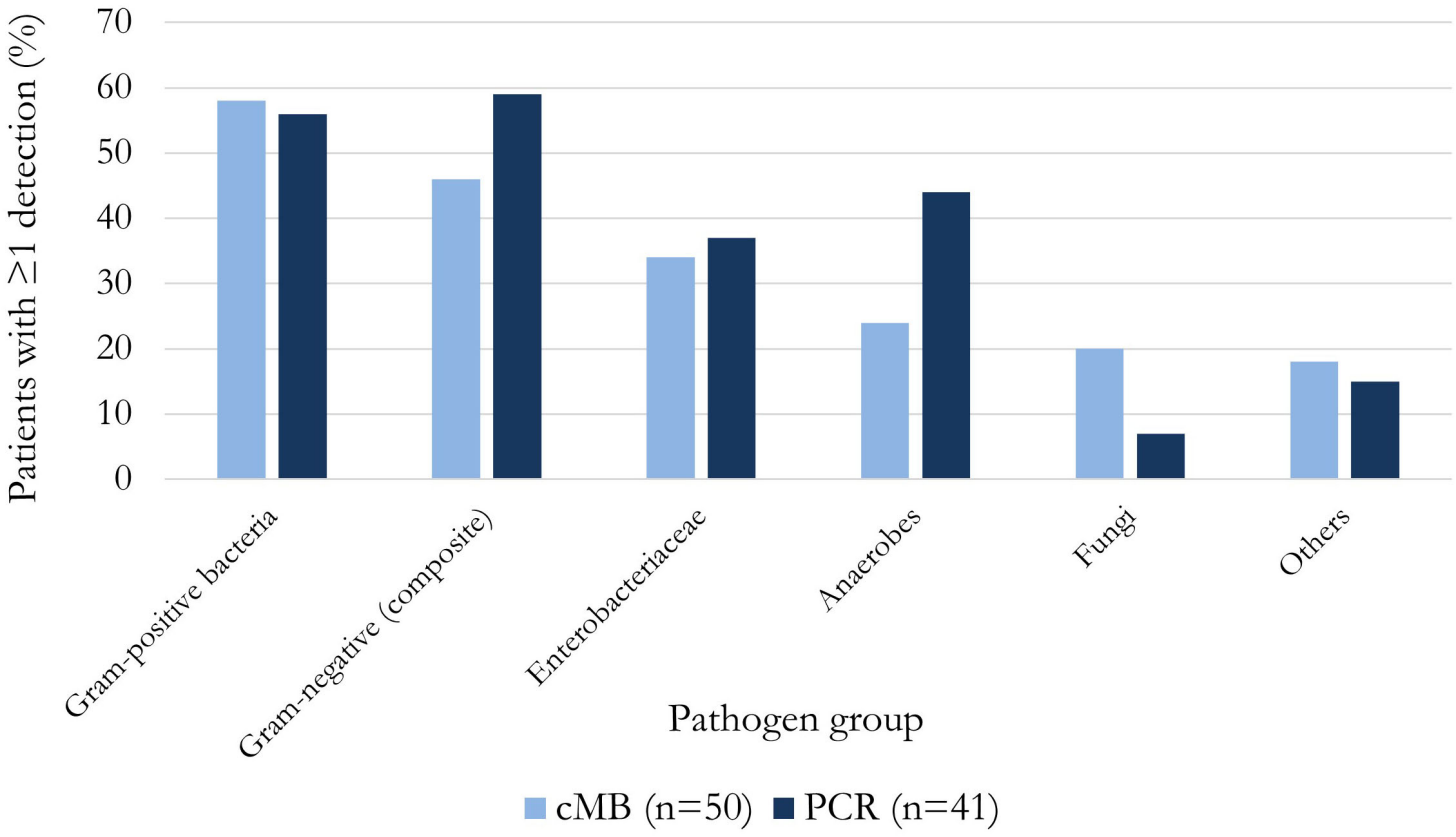
Proportion of patients with detected pathogens by group by diagnostic method (cMB, n=50; PCR, n=41). Non-fermenting bacteria were not detected and are therefore not shown.

The mean number of pathogens per sample was 2.08 (cMB, n=50) and 2.6 (PCR, n=41). Negative results were observed in 34% (17/50) of cMB samples and 27% (11/41) of PCR samples. Single-pathogen detections occurred in 18% (cMB, 9/50) and 17% (PCR, 7/41) (**Figure 1**). Samples with ≥3 pathogens were observed in 32% (16/50) of cMB samples and 48% (20/41) of PCR samples. Samples with ≥5 detected organisms were also observed more often in PCR samples (17%; 7/41) than in cMB samples (12%; 6/50) (**Figure 1**). Overall detection rates of major pathogen groups are summarized in **Table 5**. Gram-positive bacteria were detected in 58% of samples by cMB (29/50) and in 56% of samples with valid PCR results (23/41). Aerobic/facultative Gram-negative bacteria were detected in 46% of samples by cMB (23/50) and in 59% of samples with valid PCR results (24/41). Within this group, *Enterobacteriaceae* were the predominant subgroup and were detected in 34% of the samples by cMB (17/50) and in 37% of samples with valid PCR results (15/41). Anaerobic bacteria were numerically more frequently detected by PCR (44%, 18/41) than by cMB (24%, 12/50). As shown in **Table 5**, anaerobic detections were mainly attributable to the *Bacteroides fragilis* group and the combined *Bacteroides* spp./*Prevotella* spp. target. The *Bacteroides fragilis* group was detected in 6 cMB samples (12%; 6/50) and in 11 PCR samples (27%; 11/41), whereas *Bacteroides* spp./*Prevotella* spp. were detected in 9 cMB samples (18%; 9/50) and in 18 PCR samples (44%; 18/41). Fungal pathogens were identified more often by cMB (20%, 10/50) than by PCR (7%, 3/41) (**Figure 2**). This difference was mainly driven by *Candida albicans*, which was detected in 7 cMB samples (14%; 7/50) but only in one PCR sample (2%; 1/41). In contrast, *Candida glabrata* was detected in 2 PCR samples (5%; 2/41), whereas no *Candida glabrata* detections were reported by cMB.

**Table 5:** Pathogens detected by conventional microbiological culture (cMB) (n=50) and multiplex PCR (n=41).

Pathogen	cMB (n=50): detections, n; patients, n (%)	PCR (n=41): detections, n; patients, n (%)
Gram-positive bacteria	**Detections: 38** **Patients: 29 (58%)**	**Detections: 42** **Patients: 23 (56%)**
Coagulase-negative staphylococci	7	9
*Enterococcus faecalis*	11	8
*Enterococcus* spp.	8	14
*Streptococcus* spp.	11	8
*Staphylococcus aureus*	1	3
*Enterobacteriaceae*	**Detections: 21** **Patients: 17 (34%)**	**Detections: 23** **Patients: 15 (37%)**
*Escherichia coli*	10	12
*Klebsiella aerogenes*	0	0
*Enterobacter cloacae*	3	2
*Klebsiella pneumoniae*	4	4
*Klebsiella oxytoca*	1	2
*Klebsiella variicola*	0	0
*Proteus* spp.	3	3
Anaerobes / facultative anaerobes	**Detections: 15** **Patients: 12 (24%)**	**Detections: 29** **Patients: 18 (44%)**
*Aeromonas* spp.	0	0
*Bacteroides fragilis* group	6	11
*Bacteroides* spp. */ Prevotella* spp.	9	18
Non-fermenting bacteria	**Detections: 0** **Patients: 0 (0%)**	**Detections: 0** **Patients: 0 (0%)**
*Acinetobacter baumannii*	0	0
*Pseudomonas aeruginosa*	0	0
Fungi	**Detections: 10** **Patients: 10 (20%)**	**Detections: 6** **Patients: 3 (7%)**
*Candida* spp.	2	3
*Candida albicans*	7	1
*Candida glabrata*	0	2
*Candida tropicalis*	1	0
*Candida krusei*	0	0
Others	**Detections: 20** **Patients: 9 (18%)**	**Detections: 6** **Patients: 6 (15%)**

Absolute numbers represent the total number of pathogen or target detections. Percentages indicate the proportion of patients with at least one detection of the respective pathogen group or target, using the respective diagnostic cohort as denominator (cMB n=50, PCR n=41). Detection counts may exceed patient counts because multiple targets may be detected in a single sample. ‘Others’ comprises universal bacterial detections and additional panel targets not assigned to the predefined major pathogen groups.

Percentages refer to the proportion of patients in whom the respective pathogen or pathogen group was detected at least once.Bold values indicate summary rows for the respective pathogen groups.

### 3.4 Resistance marker detections

Resistance marker results were available for all samples with valid PCR results (n=41). At least one resistance marker was detected in 10 samples (24%). The most frequently detected resistance markers were oxacillin resistance markers (mecA), which were identified in 5 samples. Nitroimidazole resistance markers (nimA) were detected in 2 samples. Glycopeptide resistance markers (vanB), a third-generation cephalosporin resistance marker (ctx-M), and fluoroquinolone resistance markers (qnrB) were detected in 1 sample each. The detected resistance markers and co-detected pathogens are summarized in **Table 6**. Due to the polymicrobial nature of most samples, resistance markers could not be reliably assigned to individual pathogens.

**Table 6:** Resistance markers detected by multiplex PCR in samples with valid PCR results (n=41) and co-detected pathogens.

Patient ID	Antibiotic class	Resistance marker / gene	Pathogens detected in the same PCR sample
20	Oxacillin	mecA	Coagulase-negative *staphylococci, Enterococcus faecalis, Enterococcus* spp.*, Streptococcus* spp.*, Bacteroides fragilis, Bacteroides* spp. */ Prevotella* spp.*, Escherichia coli, Finegoldia magna, Clostridium perfringens*
33	Oxacillin	mecA	Coagulase-negative *Staphylococcus, Staphylococcus aureus, Bacteroides* spp. */ Prevotella* spp.
36	Oxacillin	mecA	Coagulase-negative *Staphylococcus, Enterococcus faecalis, Enterococcus* spp.*, Staphylococcus aureus, Bacteroides* spp. */ Prevotella* spp.
44	Oxacillin	mecA	Coagulase-negative *Staphylococcus, Streptococcus* spp., *Escherichia coli*
49	Oxacillin	mecA	Coagulase-negative *Staphylococcus, Enterococcus faecalis, Enterococcus* spp.*, Streptococcus* spp.*, Klebsiella pneumoniae, Candida* spp., *Candida albicans, Candida glabrata*
15	Glycopeptides	vanB	*Enterococcus* spp.*, Candida* spp.*, Candida glabrata*
47	Cephalosporins (3^rd^ generation)	ctx-M	Coagulase-negative *Staphylococcus, Escherichia coli, Klebsiella pneumoniae*
8	Nitroimidazoles	nimA	*Enterococcus faecalis, Enterococcus* spp.*, Streptococcus* spp., *Bacteroides fragilis, Bacteroides* spp. */ Prevotella* spp., *Escherichia coli, Klebsiella pneumoniae, Klebsiella oxytoca, Proteus* spp.*, Finegoldia magna*
39	Nitroimidazoles	nimA	*Streptococcus* spp., *Bacteroides fragilis, Bacteroides* spp. */ Prevotella* spp.*, Proteus* spp.
17	Fluoroquinolones	qnrB	*Bacteroides fragilis, Bacteroides* spp. */ Prevotella* spp.*, Escherichia coli*

Resistance markers are shown together with pathogens detected in the same PCR sample. In polymicrobial samples, resistance markers could not be reliably assigned to individual pathogens. Therefore, listed pathogens should not be interpreted as confirmed carriers of the respective resistance gene.

### 3.5 Concordance between PCR and cMB

**Figure 3:**
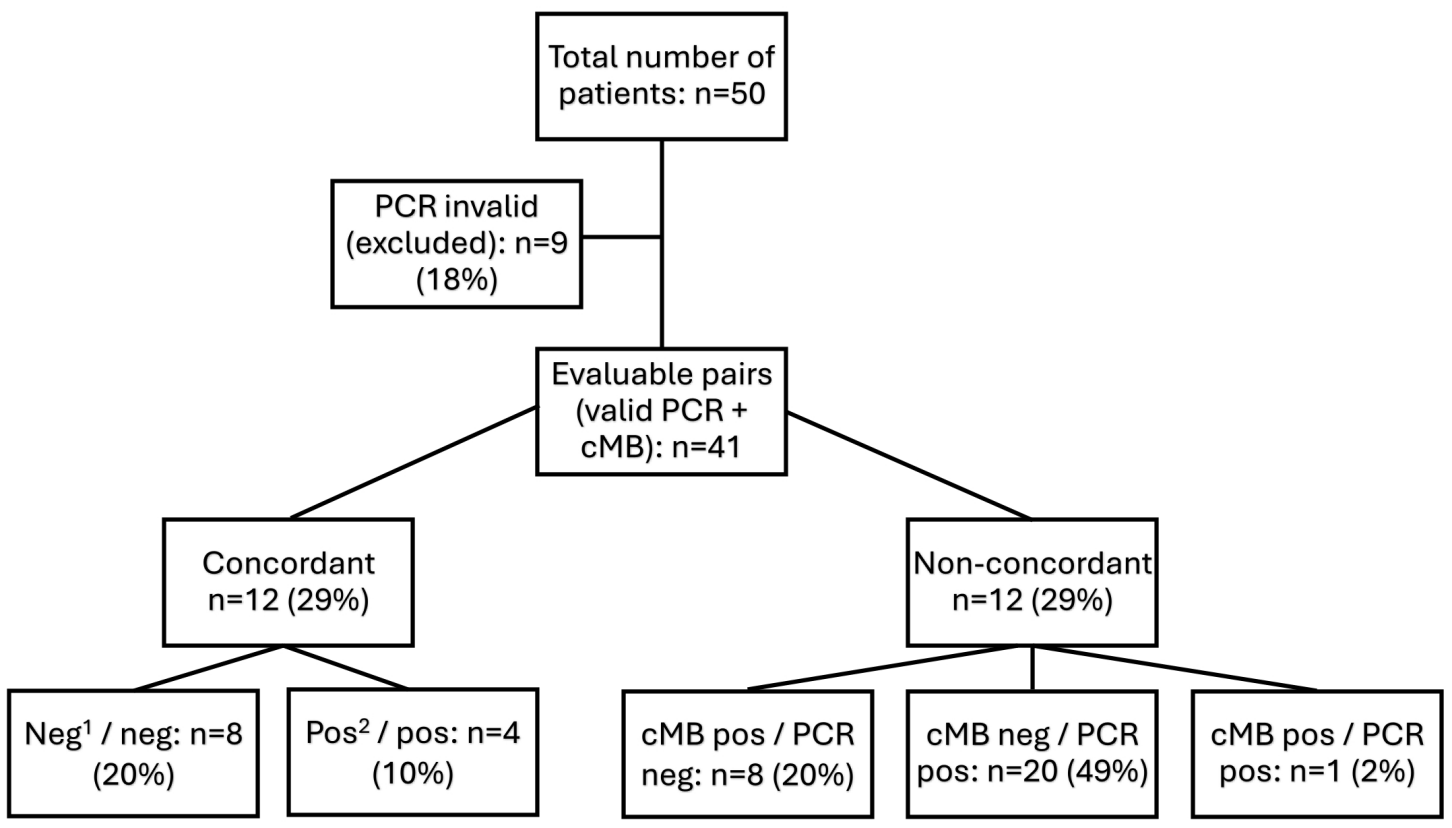
Flow diagram illustrating concordant and discordant conventional microbiological culture (cMB) and multiplex PCR (PCR) results. Percentages refer to the number of evaluable sample pairs. Complete sample-level concordance was defined as identical pathogen detection profiles between cMB and PCR. Non-concordant includes sample pairs with non-identical pathogen targets. ^1^neg, no pathogen detected; ^2^pos, ≥1 pathogen detected.

Complete concordance between PCR and cMB was observed in 12 cases (29%, 12/41), whereas discordant results were identified in 29 cases (71%, 29/41). Complete concordance at the sample level was defined as identical pathogen detection profiles between cMB and PCR within an evaluable sample pair. Within the non-concordant group, PCR detected at least one pathogen not identified by cMB in 20 samples (49%, 20/41) (**Figure 3**; **Table 7**). Since complete sample-level concordance does not reflect partial agreement for individual pathogen groups, an additional target-level concordance analysis was performed and is shown in **Table 7**. Using the definition described in the Methods section, target-level agreement rates were 82% for Gram-positive bacteria, 96% for *Enterobacteriaceae*, 85% for anaerobes/facultative anaerobes and 94% for fungi. Non-fermenting bacteria showed 100% target-level agreement because no detections occurred by either method.

**Table 7:** Target-level concordance of pathogen detection between PCR and cMB in evaluable samples (n=41), analyzed on a per-pathogen basis.

Pathogen	cMB+/PCR+	cMB+/PCR-	cMB-/PCR+	cMB-/PCR-
Gram-positive bacteria (5 pathogens × 41 samples)	**18**	**13**	**24**	**150**
Coagulase-negative Staphylococcus	4	2	5	30
*Enterococcus faecalis*	6	3	2	30
*Enterococcus* spp.	4	2	10	25
*Streptococcus* spp.	3	6	5	27
*Staphylococcus aureus*	1	0	2	38
Anaerobes / Facultative Anaerobes (3 pathogens × 41 samples)	**11**	**0**	**18**	**94**
*Aeromonas* spp.	0	0	0	41
*Bacteroides fragilis*	4	0	7	30
*Bacteroides* spp. */ Prevotella* spp.	7	0	11	23
*Enterobacteriaceae* (7 pathogens × 41 samples)	**13**	**2**	**10**	**262**
*Escherichia coli*	8	0	4	29
*Enterobacter aerogenes*	0	0	0	41
*Enterobacter cloacae*	1	1	1	39
*Klebsiella pneumoniae*	2	1	2	36
*Klebsiella oxytoca*	0	0	2	39
*Klebsiella variicola*	0	0	0	41
*Proteus* spp.	2	0	1	38
Non-fermenting Bacteria (2 pathogens × 41 samples)	**0**	**0**	**0**	**82**
*Acinetobacter baumannii*	0	0	0	41
*Pseudomonas aeruginosa*	0	0	0	41
Fungi (5 pathogens × 41 samples)	**1**	**8**	**5**	**191**
*Candida* spp.	0	2	3	36
*Candida albicans*	1	5	0	35
*Candida glabrata*	0	0	2	39
*Candida tropicalis*	0	1	0	40
*Candida krusei*	0	0	0	41

Values represent target-level comparisons between cMB and PCR in the 41 evaluable sample pairs. cMB+/PCR+ indicates concordant positive detections, cMB+/PCR- indicates cMB-only detections, cMB-/PCR+ indicates PCR-only detections and cMB-/PCR- indicates concordant negative target comparisons. Group rows summarize all target-level comparisons within the respective pathogen group; therefore, totals correspond to the number of targets multiplied by 41 samples. Individual samples may contribute more than one detection or target comparison.Bold values indicate summary rows for the respective pathogen groups.

### 3.6 Pathogen distribution by anatomical sampling site

To further characterize diagnostic findings in relation to anatomical localization, microbiological results were analyzed according to the anatomical sampling site (upper gastrointestinal sites, n=36; lower abdominal/colorectal sites, n=14;

**Table 4**). By cMB, negative results were observed in 42% of upper abdominal samples (15/36) and in 14% of lower abdominal/colorectal samples (2/14). Among samples with valid PCR results, negative findings were observed in 33% of upper abdominal samples (10/30) and in 18% of lower abdominal/colorectal samples (2/11). Overall, negative results occurred more frequently in upper abdominal than in lower abdominal/colorectal samples for both diagnostic methods. These site-specific findings are descriptive given the smaller number of lower abdominal/colorectal samples.

**Figure 4:**
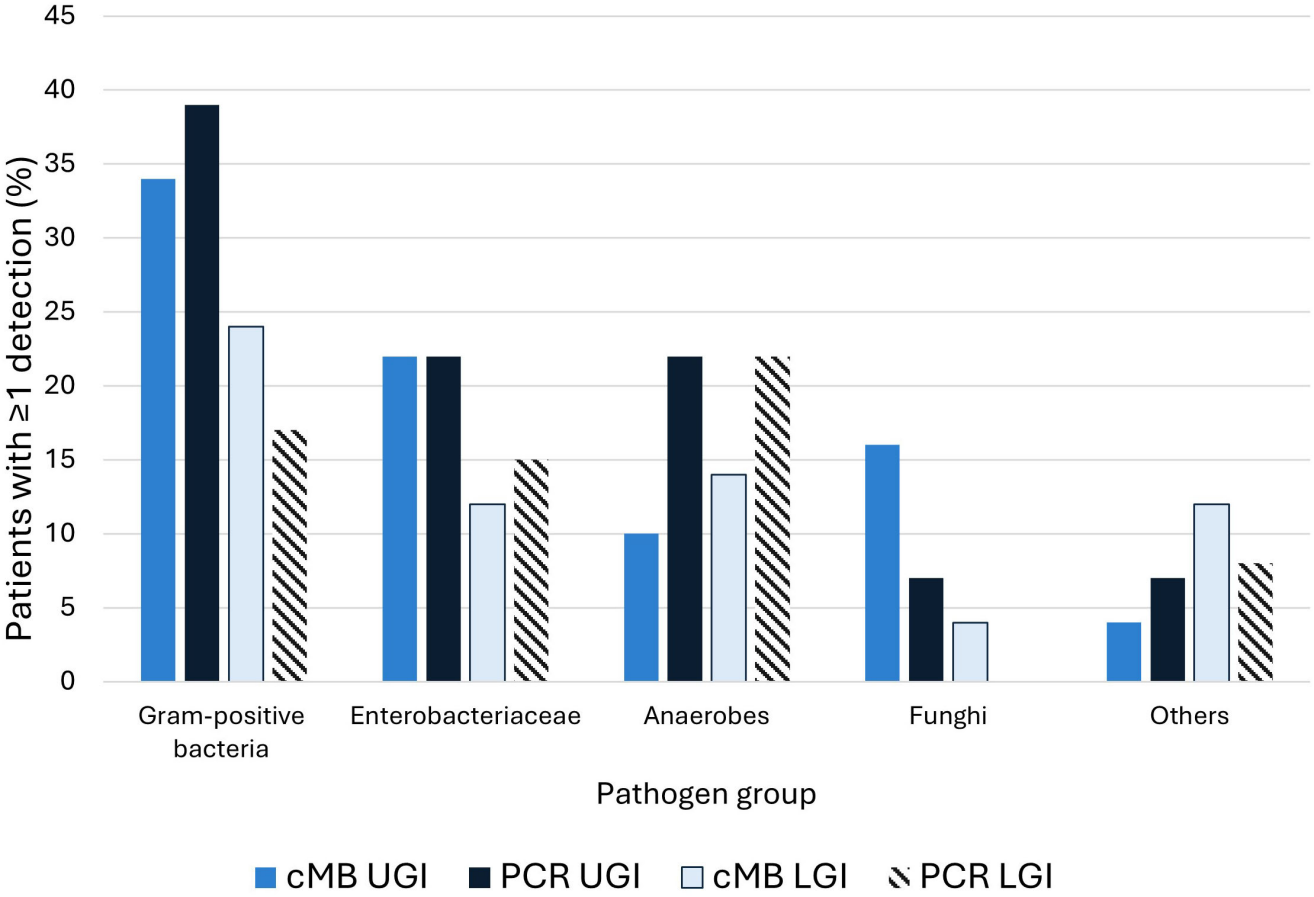
Proportion of patients with at least one detection of the indicated pathogen groups in upper abdominal sites (UA) and lower abdominal/colorectal sites (LA) by conventional microbiological culture (cMB: UA n=36, LA n=14) and multiplex PCR (PCR valid: UA n=30, LA n=11). Non-fermenting bacteria were not detected and are therefore not shown.

In samples with positive microbiological findings, the distribution of major pathogen groups differed between upper abdominal and lower abdominal/colorectal sites (**Figure 4**). Gram-positive bacteria were detected in a substantial proportion of patients in both upper abdominal and lower abdominal/colorectal samples by cMB and PCR, with higher detection rates observed in upper abdominal samples. *Enterobacteriaceae* were identified at comparable frequencies in upper abdominal and lower abdominal/colorectal samples by both diagnostic methods. In contrast, anaerobic bacteria were detected more frequently in lower abdominal/colorectal samples than in upper abdominal samples, particularly by multiplex PCR. Fungal pathogens were predominantly detected in upper abdominal samples, whereas detections in lower abdominal/colorectal samples were less frequent. No non-fermenting bacteria were identified in either anatomical site by either diagnostic method.

## 4 Discussion

In this prospective observational study, we assessed the diagnostic performance and potential clinical applicability of a multiplex PCR system (Unyvero A50 system) compared with cMB in patients with suspected hollow organ perforation and IAI. Our findings indicate that multiplex PCR provides substantially shorter diagnostic turnaround times while offering complementary information on pathogen detection, particularly for anaerobic bacteria. However, clinical outcome effects and PCR-guided antimicrobial treatment changes were not assessed.

### 4.1 Turnaround time

The markedly shorter turnaround time observed for multiplex PCR compared with conventional microbiological culture underscores the potential of molecular diagnostics to accelerate pathogen identification in IAIs (Arvaniti et al., 2022). In the present study, PCR results were available on the day of surgery or the following day in all cases with valid PCR results, whereas culture-based diagnostics were reported ≥2 days after sample collection in all cases. This time advantage is clinically relevant, as delays in microbiological results frequently require empiric broad-spectrum antimicrobial therapy, which may be associated with increased toxicity, selection of resistant organisms and prolonged treatment duration (Candel et al., 2024; Sartelli et al., 2024). Earlier availability of diagnostic information may therefore support earlier diagnostic confirmation and antimicrobial reassessment. Comparable reductions in diagnostic turnaround time using multiplex PCR systems have been reported in previous studies in patients with severe intra-abdominal infections and other critical care settings (Kunze et al., 2015; Ciesielczuk et al., 2018; Horn et al., 2024). Taken together, these studies support the role of molecular POC approaches as adjuncts to cMB rather than as stand-alone replacements (Candel et al., 2024).

### 4.2 Pathogen detection and polymicrobial findings

While both methods yielded negative and low-pathogen-count results in a substantial proportion of samples, PCR more often detected polymicrobial findings. As described in the Methods section, this may partly reflect overlapping genus- and species-level targets (e.g., *Enterococcus* spp. and *E. faecalis*) rather than additional distinct organisms. These findings suggest that multiplex PCR may provide additional descriptive information on polymicrobial IAIs, particularly by increasing detections of anaerobic and other fastidious organisms, consistent with previous comparative studies (Ciesielczuk et al., 2018; Kakizaki et al., 2023; Horn et al., 2024).

### 4.3 Concordance between PCR and cMB

Regarding diagnostic concordance, complete sample-level concordance between PCR and cMB was observed in 29% of evaluable samples. This relatively low rate reflects the strict definition of complete sample-level concordance as identical pathogen detection profiles between both methods within an evaluable sample pair. In the remaining 71% of cases, discordant findings were identified. In nearly half of the evaluable samples (49%), PCR detected at least one pathogen that was not identified by cMB. Because this strict sample-level definition does not capture partial agreement within individual pathogen groups, target-level agreement was additionally assessed descriptively. Target-level agreement was 82% for Gram-positive bacteria, 96% for Enterobacteriaceae, 85% for anaerobes/facultative anaerobes and 94% for fungi. These values should be interpreted cautiously, as they are strongly influenced by concordant negative target comparisons and therefore do not necessarily reflect agreement for clinically relevant positive findings.

### 4.4 Discordant findings and anaerobic detections

The pattern of discordance differed between pathogen groups. PCR-only detections were particularly prominent among anaerobic targets, mainly involving the *Bacteroides fragilis* group and the combined *Bacteroides* spp./*Prevotella* spp. target. This observation is biologically plausible, as *Bacteroides* and *Prevotella* species are common constituents of the intestinal microbiota and clinically relevant pathogens in secondary peritonitis. Strict anaerobes are particularly susceptible to preanalytical factors such as oxygen exposure, transport conditions, and delays in sample processing. Since conventional culture depends on viable organisms, these factors may reduce culture-based recovery. In contrast, PCR detects microbial DNA and is therefore less dependent on organism viability. Similar patterns have been reported in comparative studies of molecular diagnostics and culture, supporting the potential diagnostic value of PCR for oxygen-sensitive or fastidious organisms (Ciesielczuk et al., 2018; Kakizaki et al., 2023; Horn et al., 2024).

PCR-only detections should be interpreted cautiously. PCR may detect DNA from non-viable organisms, particularly after antimicrobial exposure, and does not prove the presence of viable pathogens. Prior antimicrobial therapy before intraoperative sampling may represent a plausible explanation for some PCR-positive/cMB-negative findings. Reliable data on antimicrobial administration before or during surgery and the exact timing relative to intraoperative sample collection were not systematically available in our cohort. Therefore, the influence of antimicrobial pretreatment on PCR/cMB discordance could not be assessed.

In addition, as the peritoneal cavity is normally sterile, these findings should not be described as peritoneal colonization. Rather, they may reflect true infection, inoculation of the peritoneal cavity from colonized hollow viscera after perforation, detection of DNA from non-viable organisms, or findings of uncertain clinical relevance in polymicrobial samples. Accordingly, the added value of multiplex PCR lies primarily in providing complementary microbiological information that needs to be interpreted in conjunction with clinical findings, antimicrobial pretreatment, the anatomical source of infection and adequacy of source control (Candel et al., 2024).

### 4.5 Fungal detections

In contrast to anaerobic bacteria, fungal pathogens were detected less frequently by PCR than by cMB, particularly for *Candida albicans*. This finding illustrates that molecular testing did not uniformly increase pathogen detection across all pathogen groups. In the absence of an independent reference standard, the reasons for this discrepancy remain uncertain. Possible explanations include low fungal burden in peritoneal samples, fungal DNA extraction challenges due to the fungal cell wall, assay-related sensitivity limitations for individual *Candida* species and differences in detection principles between culture and PCR. Conversely, culture-positive *Candida* findings in polymicrobial peritoneal samples do not necessarily prove invasive intra-abdominal candidiasis. Therefore, fungal results from either method should be interpreted cautiously and in conjunction with host risk factors, clinical findings and adequacy of source control (Bassetti et al., 2013; Ciesielczuk et al., 2018; Xie et al., 2022; Horn et al., 2024).

### 4.6 Resistance marker detections

Resistance marker detection may provide early information on selected resistance mechanisms before phenotypic susceptibility testing becomes available. In the present cohort, at least one resistance marker was detected in 10 of 41 valid PCR samples. However, interpretation of resistance markers in IAIs is challenging because samples are frequently polymicrobial. In such settings, resistance genes cannot be reliably assigned to a specific organism. Moreover, the PCR panel includes only selected resistance determinants and does not provide comprehensive phenotypic susceptibility testing. Therefore, PCR-based resistance marker detection should be interpreted as supplementary early information and cannot replace conventional culture-based antimicrobial susceptibility testing. Similar limitations in resistance marker interpretation have been reported for other Unyvero applications. In a pediatric and neonatal pneumonia cohort, Papan et al. observed variable performance depending on pathogen group and reported limitations in the interpretation of resistance gene detections compared with phenotypic susceptibility testing (Papan et al., 2018).

### 4.7 Comparison with previous Unyvero IAI and multiplex PCR studies

Previous studies evaluating the Unyvero IAI system and related multiplex PCR approaches in intra-abdominal infections have reported heterogeneous results. Ciesielczuk et al. demonstrated that the Unyvero IAI cartridge can provide rapid pathogen detection from intra-abdominal samples and may identify additional microorganisms compared with conventional culture, while also showing discordant results between both methods (Ciesielczuk et al., 2018). In a clinical setting, Horn et al. reported reduced turnaround times and additional pathogen detections using PCR-based microbiological testing in intra-abdominal infections, supporting the potential role of molecular diagnostics as a rapid adjunct to culture-based workflows (Horn et al., 2024). Similarly, Kakizaki et al. described rapid bacterial identification by multiplex PCR in acute abdominal infections (Kakizaki et al., 2023). Recent data by Schwab et al. evaluating the Unyvero IAI system in patients with peritoneal dialysis- and liver cirrhosis-related peritonitis also demonstrated a clear time advantage of PCR compared with culture (Schwab et al., 2025). However, in contrast to our cohort, Schwab et al. reported lower pathogen detection rates by PCR than by culture, particularly for Gram-positive organisms. This difference may reflect differences in patient population, sample type, and expected pathogen spectrum. Whereas peritoneal dialysis- and cirrhosis-related peritonitis are often non-surgical entities with a different microbiological profile, our cohort consisted of patients with suspected hollow organ perforation or secondary peritonitis, in whom polymicrobial and anaerobic infections are more frequent.

Taken together, these studies indicate that the diagnostic performance of multiplex PCR is context-dependent and should be interpreted in relation to the clinical syndrome, sample material, and expected microbial spectrum. Overall, the available evidence supports Unyvero IAI as a diagnostic adjunct rather than a replacement for conventional culture.

### 4.8 Potential clinical role

The potential clinical role of multiplex PCR is likely greatest in critically ill or high-risk patients with complicated IAIs, secondary peritonitis, sepsis or septic shock, postoperative ICU admission after emergency abdominal surgery, prior antimicrobial exposure, healthcare-associated or postoperative infection, immunosuppression, recurrent IAI, or increased risk of multidrug-resistant organisms. In these settings, same-day microbiological information may support earlier reassessment of empiric antimicrobial therapy, particularly when unexpected pathogens or resistance markers are detected.

This is consistent with recent ICU-focused literature emphasizing that rapid molecular diagnostics are most useful when integrated with antimicrobial stewardship programs, local resistance epidemiology, and conventional susceptibility testing, particularly in patients at risk for multidrug-resistant nosocomial infections (Candel et al., 2024; Marin et al., 2025). However, PCR results should not be used as a stand-alone basis for treatment decisions. They must be interpreted together with the clinical presentation, anatomical source, adequacy of source control, prior antimicrobial exposure, local resistance epidemiology, resistance marker results, and conventional culture including phenotypic susceptibility testing.

Evidence from other ICU infection syndromes suggests that rapid PCR-based diagnostics may influence antimicrobial management when embedded in structured stewardship algorithms, as shown in patients with pneumonia at risk of Gram-negative infection (Darie et al., 2022). However, such effects cannot be inferred from the present observational study and require prospective interventional evaluation in patients with IAIs.

Moreover, the present study does not establish a measurable clinical benefit. No predefined PCR-guided antimicrobial stewardship intervention was implemented, and changes in antimicrobial therapy attributable to PCR results were not systematically recorded. Accordingly, the study cannot determine whether PCR-based diagnostics led to earlier adequate therapy, antimicrobial escalation or de-escalation, reduced antimicrobial exposure, shorter ICU or hospital length of stay, or improved survival. The value described in this study should therefore be understood as additional diagnostic information rather than as proven clinical outcome benefit.

### 4.9 Limitations

Several limitations of our study should be acknowledged. First, the sample size was small, and no formal sample size calculation was performed because the study was designed as an exploratory prospective observational study. Accordingly, the results should be interpreted as descriptive and exploratory rather than confirmatory. The limited sample size precludes robust estimates of diagnostic accuracy and limits the generalizability of the findings. Second, 9 of 50 PCR runs yielded invalid results according to the manufacturer’s system output and were excluded from PCR-based and concordance analyses. This reduced the number of evaluable paired samples to 41, thereby limiting the precision and robustness of concordance analyses. In addition, a potential selection bias cannot be excluded because samples with invalid PCR results could not be included in paired comparisons. The exact technical causes of invalid PCR results were not systematically documented, as the system output did not specify the underlying cause of invalidity. The invalid PCR rate therefore represents an important practical limitation for implementation as a routine point-of-care diagnostic tool.

Furthermore, the PCR panel is limited to predefined targets and does not capture all potential pathogens. Resistance marker analysis was descriptive, and detected resistance genes could not be reliably assigned to individual pathogens in polymicrobial samples. In addition, PCR-based resistance marker detection does not replace phenotypic antimicrobial susceptibility testing. Prior antimicrobial therapy and preanalytical variability may also have affected detection rates. Reliable data on antimicrobial administration before or during surgery and exact timing relative to intraoperative sampling were not systematically available, preventing assessment of the influence of antimicrobial pretreatment on PCR/cMB discordance.

## 5 Conclusion

In conclusion, multiplex PCR may represent a useful diagnostic adjunct to conventional culture in the diagnosis of IAIs, primarily due to its rapid turnaround time and numerically more frequent detection of selected pathogen groups, particularly anaerobic bacteria, compared with culture-based methods. However, its limitations regarding pathogen coverage, fungal detection, invalid PCR results, and interpretation of resistance markers in polymicrobial samples must be considered. The present study did not assess PCR-guided treatment changes or clinical outcomes. Therefore, clinical benefit remains unproven and should be evaluated in larger multicenter prospective interventional studies.

## Data Availability

The raw data supporting the conclusions of this article will be made available by the authors, without undue reservation.
